# The Association between Serum Anion Gap and All-Cause Mortality in Cerebral Infarction Patients after Treatment with rtPA: A Retrospective Analysis

**DOI:** 10.1155/2022/1931818

**Published:** 2022-05-12

**Authors:** Hesong Wang, Chang Liu, Heng Xu, Yunan Zhang, Pengyi Gao, Shaohui Geng, Weijia Kong, Yuxing Zhi, Kai Yuan, Lichun Tian

**Affiliations:** ^1^School of Life Sciences, Beijing University of Chinese Medicine, Beijing, China; ^2^School of Acupuncture-Moxibustion and Tuina, Beijing University of Chinese Medicine, Beijing, China; ^3^Department of Oncology, Beijing Hospital of Traditional Chinese Medicine, Capital Medical University, Beijing, China; ^4^Qi-Huang Chinese Medicine School, Beijing University of Chinese Medicine, Beijing, China; ^5^Beijing Research Institute of Chinese Medicine, Beijing University of Chinese Medicine, Beijing, China

## Abstract

**Background:**

No epidemiological study has determined the association between the anion gap (AG) and all-cause mortality in cerebral infarction patients after treatment with rtPA. This study is aimed at using AG levels as a prognostic factor for evaluating cerebral infarction patients after receiving rtPA treatment and to help the resident physicians accurately evaluate the therapeutic plan of rtPA.

**Methods:**

We extracted clinical data from the public database (MIMIC-IV database V1.0) and used the Kaplan-Meier curve to estimate the survival probabilities of cerebral infarction patients after rtPA treatment for the one-year, four-year, and whole period by log-rank test in 948 intensive care unit patients. Cox proportional hazard models were used to assess the association between AG and one-year, four-year, and whole period mortality in cerebral infarction patients after treatment with rtPA.

**Results:**

Kaplan-Meier survival curve indicated a higher AG value is significantly associated with an increased risk of one-year, four-year, and whole-period all-cause mortality in cerebral infarction patients after treatment with rtPA. Model I adjusted for ethnicity, age, gender, and skin tone. Model II adjusted for ethnicity, age, gender, skin tone, hypertension, diabetes, coronary atherosclerosis, congestive heart failure, peripheral vascular, hyperlipidemia, acute myocardial infarction (AMI), respiratory failure, and end-stage renal diseaseesrd (ESRD). On the basis of model II, model III adjusted for WBC, BUN, creatinine, platelet, MCH, MCHC, MCV, RBC, and RDW. In addition, there was better predictive ability between higher AG levels and mortality in certain subgroups, such as patients with platelet ≤ 247, RBC > 3.11.

**Conclusion:**

Serum AG is positively related to all-cause mortality in cerebral infarction patients after treatment with rtPA.

## 1. Introduction

Cerebral infarction refers to the localized ischemic necrosis or softening of brain tissue caused by cerebral blood supply obstacles, ischemia, and hypoxia. It is also the most common stroke-type clinical disease. The onset of cerebral infarction is more rapid, and its fatality rate and disability rate are higher. The incidence of cerebral infarction patients under 45 years old ranges from 3.4% to 11.3% per 100,000 cases [1]. The incidence of cerebral infarction in the elderly accounts for 50% to 80% of all kinds of cerebrovascular diseases [2]. In the acute stage of cerebral infarction, timely and effective treatment is closely related to the prognosis [3]. At present, early thrombolysis under the guidance of time window is a clinically recognized treatment [4, 5], and intravenous application of recombinant tissue plasminogen activator (rtPA) is one of the most effective methods of acute cerebral infarction [6]. However, the clinical prognosis of patients after receiving rtPA therapy is also different [7, 8]. Therefore, there was an important clinical value to explore the prognostic factors of cerebral infarction patients receiving rtPA therapy.

The calculation of the serum anion gap (AG) was [Na(+)] − ([HCO(3)(−)] + [Cl(−)]), and it has a certain value in differential diagnosis in the etiology and types of metabolic acidosis [9]. Studies have shown that serum AG can be regarded as a prognostic biomarker for patients admitted to the intensive care unit [10]. The ischemic state can cause metabolic disorders in the brain, which leads to trigger changes in ion concentration [11]. The acid-base balance in the systemic circulation responds quickly to cerebral ischemic events [12], so we infer that AG may be a potential biomarker of cerebral infarction.

Previous studies have not used AG as a prognostic indicator to evaluate the prognosis of cerebral infarction patients after treatment with rtPA. Therefore, we tried to find the potential association between AG and the prognosis of cerebral infarction patients after treatment with rtPA.

## 2. Methods

### 2.1. Data Source

The data of patients is from the database of clinical data in Medical Information Mart for Intensive Care IV (MIMIC-IV) database V1.0, collected from Metavision bedside monitors [1]. The MIMIC-IV, a public database based on the real world, contains more than 60,000 ICU patients admitted to Beth Israel Deaconess Medical Center from 2008 to 2019. Our access to the database and data has been approved by the institutional review committee of the Massachusetts Institute of Technology (Cambridge, Massachusetts, USA), He-song Wang completed the Collaborative Institutional Training Initiative course named “Data or Specimens Only Research” and obtained the certification (certification number: 44627934). After becoming a credentialed user on PhysioNet and signing the data use agreement, we can work with the data.

### 2.2. Patient-Selection Criteria

Criteria for inclusion of patients:(1) cerebral infarction was diagnosed with ICD-9 and ICD-10 code at the first hospital admission; (2) first treatment with rtPA; (3) patients whose AG was measured within 48 hours after treatment with rtPA.

Criteria for exclusion of patients:(1) anion gap data missing after treatment with rtPA; (2) readmission to the hospital; (3) patients with abnormally high and low values of AG; (4) patients with other abnormal data (① The time of recording the AG value of the patient was before death. ② The patient who was given rtPA treatment was after death).

### 2.3. Data Extraction

The clinical data of all patients were obtained in MIMIC-IV database and retrieved by pgadmin 4 for post-gresql management platform by using Structured Query Language (SQL) on PostgreSQL (v9.6). The data we extracted are the clinical indicators of cerebral infarction patients after receiving rtPA treatment. The extracted data included demographics, vital signs, laboratory tests, comorbidities, and drug treatment. The extracted data specifically includes admission type, ethnicity, age, gender, hypertension, diabetes, coronary atherosclerosis, congestive heart failure, peripheral vascular, hyperlipidemia, acute myocardial infarction (AMI), respiratory failure, end-stage renal diseaseesrd (ESRD), white blood cell (WBC), blood urea nitrogen (BUN), creatinine, mean corpusular hemoglobin (MCH), mean corpusular hemoglobin concerntration (MCHC), mean corpusular volume (MCV), platelet, red blood count (RBC), red cell distribution width (RDW), international normalized ratio (INR), and anion gap (AG) (the anion gap dosage is performed after the rTPA).

### 2.4. Statistical Analysis

The mean ± standard deviation (SD) or medians and interquartile range (IQR) were used to represent continuous variables. Categorical data were presented as frequency or percentage. Based on the median of AG, the patients were divided into high AG group and low AG group. We determine the significance of the difference between the means and proportions among the study groups with the chi-square, one-way ANOVA, and Kruskal–Wallis H-tests. The Cox proportional hazard model was used to estimate the correlation between the AG and one-year, four-year, and overall survival mortality in cerebral infarction. In addition, we used the Kaplan-Meier curve to estimate the survival probabilities of cerebral infarction patients after rtPA treatment for the whole period, one-year, and four-year by log-rank test. Then, we analyze the effect of confounding factors on the model and give four adjusted models, and the results are presented as hazard ratio (HR) with 95% confidence intervals (CI). We selected the fully adjusted confounders with more than 10% change in the effect estimate [13]. Crude model adjusted for none. Model I adjusted for basic characteristics of the population like ethnicity, age, gender, and skin tone. To consider the effect of complications after rtPA treatment, they were incorporated into model II. On the basis of model I, model II adjusted for complications including hypertension, diabetes, coronary atherosclerosis, congestive heart failure, peripheral vascular, hyperlipidemia, acute myocardial infarction (AMI), respiratory failure, and ESRD. On the basis of model II, the factors of relevant clinical observation indexes are added to model III, such as WBC, BUN, creatinine, platelet, MCH, MCHC, MCV, RBC, and RDW. Logistic regression models were used for subgroup analysis in Table 1, according to age strata (≤66, >66), gender, ethnicity, AMI, BUN, creatinine, INR, MCHC, MCH, platelet, PT, RBC, RDW, WBC congestive heart failure, coronary atherosclerosis, diabetes, ESRD, hyperlipidemia, hypertension, and respiratory failure. The *P* for interaction was derived from a multivariable logistic regression model. All probability values were two-sided, and *P* for interaction < 0.05 was considered statistically significant. Python (https://www.python.org/) and STATA version 15.0 were used for all statistical analyses.

## 3. Results

### 3.1. Subject Characteristics

**Figure 1:**
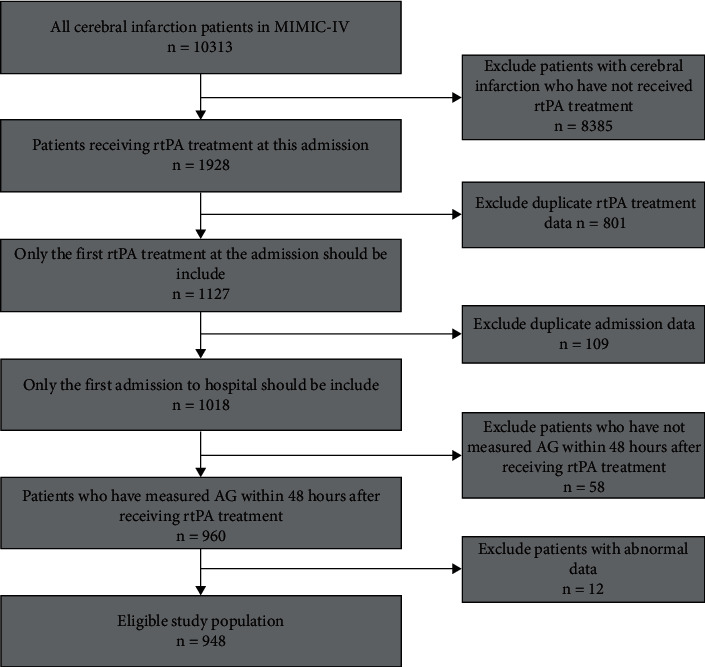
Flowchart of subject screening. Abbreviations: AG: anion gap; rtPA: recombinant tissue plasminogen activator.

The process of patient selection and exclusion is shown in Figure 1, and 948 patients fulfilled all screening criteria and were enrolled in this study. As shown in Table 2, these patients are divided into two groups according to the median of AG value. There were 503 patients in the Q1 (AG ≤ 14) group and 445 patients in the Q2 (AG > 14) group. The patients with a higher AG (AG > 14) were more likely to report some comorbidities of coronary atherosclerosis, congestive heart failure, and ESRD. The WBC along with BUN and creatinine was significantly higher in those with high serum AG values (AG > 14) than in those with low serum AG values (AG ≤ 14).

### 3.2. Relationship between AG Levels and All-Cause Mortality in Different Models

**Figure 2:**
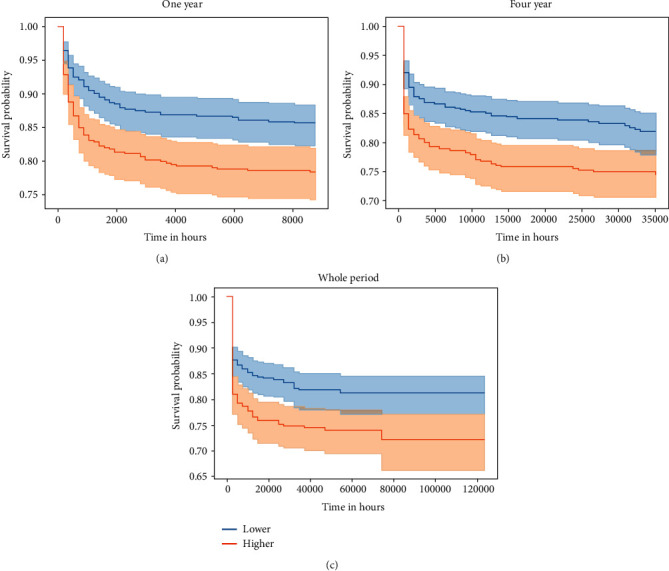
Kaplan-Meier survival curves show the relationship between AG levels and mortality in cerebral infarction patients after treatment with rtPA within one-year (a), four-year (b), and whole-period (c).

To determine the relationship between AG levels and the patient's all-cause mortality, we used the Cox proportional hazards regression model to evaluate the relationship between AG and all-cause mortality in cerebral infarction patients after treatment with rtPA. As shown in Figure 2, the Kaplan-Meier survival curve was used to show the relationship between AG levels and mortality in cerebral infarction patients after treatment with rtPA, which indicated a higher AG value is significantly associated with an increased risk of death in patients within one-year, four-year, and whole-period (*P* for whole-period = 0.0017; *P* for one-year = 0.0130; *P* for four-year = 0.0004). Then, we showed the relationship between AG levels and all-cause mortality in different models, and nonadjusted model (crude model) and adjusted models (model I, model II, model III) were shown in Table 3. The crude model adjusted for none; on the basis of crude model, model I adjusted for age, gender, ethnicity, and skin tone; on the basis of model I, model II adjusted for congestive heart failure, coronary atherosclerosis, diabetes, ESRD, AMI, hyperlipidemia, hypertension, and respiratory failure; on the basis of model II, model III adjusted for creatinine, INR, MCHC, MCH, platelet, RBC, RDW, and WBC. We found that high levels of AG (AG > 14) were associated with an increased risk of whole period all-cause mortality (crude model: HR = 1.56, 95%CI = 1.18 − 2.06, *P* = 0.002; model I: HR = 1.62, 95%CI = 1.21 − 2.15, *P* = 0.001; model II: HR = 1.56, 95%CI = 1.16 − 2.09, *P* = 0.003; model III: HR = 1.47, 95%CI = 1.08 − 2.02, *P* = 0.016), one-year all-cause mortality (crude model: HR = 1.47, 95%CI = 1.08, 2.00, *P* = 0.014; model I: HR = 1.61, 95%CI = 1.17 − 2.22, *P* = 0.003; model II: HR = 1.82, 95%CI = 1.29 − 2.56, *P* = 0.001; model III: HR = 2.18, 95%CI = 1.50 − 3.16, *P* < 0.001), and four-year all-cause mortality (crude model: HR = 1.65, 95%CI = 1.24 − 2.18, *P* = 0.001; model I: HR = 1.77, 95%CI = 1.33 − 2.36, *P* < 0.001; model II: HR = 1.68, 95%CI = 1.25 − 2.25, *P* = 0.001; model III: HR = 1.52, 95%CI = 1.10 − 2.08, *P* = 0.01).

### 3.3. Subgroup Analyses

The subgroup analysis of the relationship between AG and whole period mortality was displayed in Table 1. The tests for interactions were not statistically significant for admission, age, AMI, BUN, congestive heart failure, coronary atherosclerosis, creatinine, diabetes, ESRD, hypertension, INR, MCHC, MCH, RDW, respiratory failure, and WBC (*P* for interaction = 0.211, 0.069, 0.259, 0.461, 0.160, 0.761, 0.696, 0.324, 0.931, 0.611, 0.136, 0.181, 0.343, 0.344, 0.674, and 0.721). However, the tests for interactions were significant for ethnicity (HR = 1.56, 95%CI = 0.98 − 2.49, *P* for interaction = 0.042), gender (HR = 1.68, 95%CI = 1.12 − 2.52, *P* for interaction = 0.015), hyperlipidemia (HR = 1.21, 95%CI = 0.84 − 1.75, *P* for interaction = 0.012), platelet (HR = 1.28, 95%CI = 0.89 − 1.84, *P* for interaction < 0.001), and RBC (HR = 1.60, 95%CI = 1.11 − 2.32, *P* for interaction = 0.001). Among these strata, we found that patients with AG > 14 exhibited significantly higher mortality with platelet ≤ 247, RBC > 3.11, and a similar trend was observed in white patients and male patients. In addition, the positive association between AG and whole-period mortality was more obvious in cerebral infarction patients after treatment with rtPA without hyperlipidemia (HR = 2.19, 95%CI = 1.40 − 3.40) than those with hyperlipidemia (HR = 1.21, 95%CI = 0.84 − 1.75, *P* for interaction = 0.012).

## 4. Discussion

The serum anion gap is often helpful in diagnosing the type of disease, and in patients with metabolic acidosis, AG can classify the disease as either a normal anion gap or an elevated anion gap [9]. Various disorders in which metabolic acidosis occurs may have different effects on the serum anion gap. The most common cause of an elevated serum anion gap is the accumulation of organic or inorganic anions in metabolic acidosis, and this change is especially pronounced when acid is overproduced or excretion is reduced. There are several pathophysiological factors that can alter the serum anion gap. For every 10 g/L decrease in serum albumin concentration, the AG value decreases by about 2.3 mmol/L [14]. Accumulation of cationic paraproteins, bromides, or iodides in serum can reduce the anion gap or even make it negative. Conversely, accumulation of anion paraproteins or the development of hyperphosphatemia increases the serum anion gap, and most cases with very high serum anion gaps (i.e., values > 45 mmol/l) are associated with severe hyperphosphatemia [14, 15]. In addition, the disturbances in brain metabolism occur when there are instabilities in cerebral blood flow (CBF), which causes shifts in ion concentrations. Some studies have found that changes in cerebrovascular hemodynamics often lead to changes in acid-base balance [12], so the AG values in the brain of patients with cerebrovascular diseases often show abnormality.

The serum anion gap is a calculated entity that has been used for more than 40 years to assess acid-base disorders [14], and its greatest utility in the differential diagnosis of metabolic acidosis has been found [16, 17]. AG reflects the unmeasured anion concentration, suggesting that AG could be a potential biomarker for cerebral infarction [10]. The current research has found that high AG was associated with an increased risk of all-cause mortality, and AG could be an independent short-term prognostic factor for cerebral infarction [18]. In current clinical practice, there is still a lack of biochemical indicators to predict the mortality of cerebral infarction patients after receiving rtPA treatment. Therefore, our research aims to use AG levels as a prognostic factor for evaluating cerebral infarction patients after receiving rtPA treatment and to help resident physicians accurately evaluate the rtPA therapeutic plans.

Recent studies have shown that levels of AG were related to the prognosis of coronary artery disease, sepsis, acute kidney injury and chronic kidney disease, disseminated intravascular coagulation, acute pancreatitis, cardiogenic shock, etc. [19–24].

In this study, we found the association between serum AG value all-cause mortality in cerebral infarction patients after treatment with rtPA. First, we found patients with higher AG (AG > 14) were more likely to report some comorbidities of coronary atherosclerosis, congestive heart failure, and ESRD. The WBC along with BUN and creatinine was significantly higher in those with high serum AG values (AG > 14) than in those with low serum AG values (AG ≤ 14). Second, Kaplan-Meier survival curve indicated a higher AG value is significantly associated with an increased risk of one-year, four-year, and whole-period all-cause mortality in cerebral infarction patients after treatment with rtPA (*P* for whole-period = 0.0017; *P* for one-year = 0.0130; *P* for four-year = 0.0004). In addition, we adjusted the crude model to more effectively predict the relationship between AG level and mortality. Model I adjusted for age, gender, ethnicity, and skin tone, which is more effective in predicting AG levels and whole period (HR = 1.62, 95%CI = 1.21 − 2.15, *P* = 0.001) and four-year (HR = 1.77, 95%CI = 1.33 − 2.36, *P* < 0.001) all-cause mortality in cerebral infarction patients after treatment with rtPA. Model III is more effective in predicting AG levels and one-year (HR = 2.18, 95%CI = 1.50 − 3.16, *P* < 0.001) all-cause mortality. Finally, we found that patients with AG > 14 exhibited significantly higher mortality with platelet ≤ 247, RBC > 3.11, and a similar trend was observed in white patients and male patients. In addition, the positive association between AG and whole-period mortality was more obvious in cerebral infarction patients after treatment with rtPA without hyperlipidemia (HR = 2.19, 95%CI = 1.40 − 3.40) than those with hyperlipidemia (HR = 1.21, 95%CI = 0.84 − 1.75, *P* for interaction = 0.012).

Our study found that within 48 hours after rtPA treatment in patients with cerebral infarction, patients with AG > 14 had significantly higher overall, 1-year, and 4-year mortality than those with AG < 14. In patients with cerebral infarction, increased AG value is an important risk factor within 48 hours after rtPA treatment. We suggest that cerebral infarction patients with an AG value >14 within 48 hours after receiving rtPA treatment need follow-up detection and active treatment within 1 year, so as to reduce 1-year mortality. Therefore, serum AG value may facilitate clinical assessment of the mortality risk for cerebral infarction patients after treatment with rtPA, which is a low-cost and easily available biomarker.

## 5. Limits

**(**1) Patient data are from a single center. (2) The number of studies with the number of patients per hour was limited, and we did not analyze the predictive power of hourly AG values after rtPA treatment. (3) This is a retrospective study, and it is difficult to obtain data by blinding and randomization

## 6. Conclusion

Serum AG is positively related to all-cause mortality in cerebral infarction patients after treatment with rtPA.

## Figures and Tables

**Table 1:** Subgroup analysis of the relationship between AG and whole-period mortality.

Characteristic	*N*	HR (Q1, AG ≤ 14)	HR (Q2, AG > 14)	*P* for interaction
Admission				0.211
EW EMER	434	Ref	1.81 (1.23, 1.26)	
Other	514	Ref	1.30 (0.86, 1.97)	
Age				0.069
≤66	482	Ref	1.81 (1.24, 2.62)	
>66	466	Ref	1.32 (0.86, 2.01)	
AMI				0.259
No	937	Ref	0.58 (0.08, 4.14)	
Yes	11	Ref	1.58 (1.19, 2.09)	
BUN				0.461
≤21	462	Ref	1.29 (1.21, 2.75)	
>21	486	Ref	1.75 (1.12, 2.75)	
Congestive heart failure				0.160
No	620	Ref	1.56 (0.97, 2.53)	
Yes	328	Ref	1.56 (1.10, 2.20)	
Coronary atherosclerosis				0.761
No	666	Ref	1.54 (0.95,2.49)	
Yes	282	Ref	1.54 (1.09.2.18)	
Creatinine				0.696
≤0.9	502	Ref	1.59 (1.10, 2.30)	
>0,9	446	Ref	1.33 (0.84, 2.11)	
Diabetes				0.324
No	555	Ref	1.54 (1.01, 2.34)	
Yes	393	Ref	1.54 (1.01, 2.34)	
ESRD				0.931
No	883	Ref	1.69 (0.49, 5.82)	
Yes	65	Ref	1.52 (1.14, 2.04)	
Ethnicity				0.042
White race	584	Ref	1.57 (1.10, 2.23)	
Other	364	Ref	1.56 (0.98, 2.49)	
Gender				0.015
Female	474	Ref	1.44 (0.98, 2.11)	
Male	474	Ref	1.68 (1.12, 2.52)	
Hyperlipidemia				0.012
No	545	Ref	2.19 (1.40, 3.40)	
Yes	403	Ref	1.21 (0.84, 1.75)	
Hypertension				0.611
No	774	Ref	1.08 (0.55, 2.09)	
Yes	174	Ref	1.70 (1.24, 2.32)	
INR				0.136
≤1.25	319	Ref	1.67 (1.19, 2.35)	
>1.25	319	Ref	1.61 (1.13, 2.32)	
MCHC				0.181
≤32	475	Ref	1.54 (1.05, 2.27)	
>32	460	Ref	1.60 (1.07, 2.40)	
MCH				0.343
≤29.5	481	Ref	1.48 (0.99, 2.20)	
>29.5	454	Ref	1.66 (1.12, 2.46)	
Platelet				<0.001
≤247	468	Ref	2.09 (1.34, 3.27)	
>247	466	Ref	1.28 (0.89, 1.84)	
RBC				0.001
≤3.11	470	Ref	1.58 (1.03, 2.41)	
>3.11	465	Ref	1.60 (1.11, 2.32)	
RDW				0.344
≤15.7	475	Ref	1.50 (1.06, 2.12)	
>15.7	460	Ref	1.78 (1.10, 2.87)	
Respiratory failure				0.674
No	578	Ref	1.84 (1.22,2,78)	
Yes	370	Ref	1.32 (0.90,1.94)	
WBC				0.721
≤9.9	470	Ref	1.57 (1.08,2.28)	
>9.9	465	Ref	1.46 (0.95,2.24)	

Abbreviations: HR: hazard ratio; SD: standard deviation; AMI: acute myocardial infarction; ESRD: end-stage renal diseaseesrd; WBC: white blood cell; BUN: blood urea nitrogen; MCH: mean corpusular hemoglobin; MCHC: mean corpusular hemoglobin concentration; MCV: mean corpusular volume; RBC: red blood count; RDW: red cell distribution width; Ref: reference; Q1: quartile 1; Q2: quartile 2; Ew emer: emergency ward.

**Table 2:** Baseline characteristics of participants according to anion gap (*N* = 948).

Characteristic	Anion gap, mmol/L				*P* value
	Missing	Overall	Q1 (AG ≤ 14)	Q2 (AG > 14)	
*All patients*		948	503	445	
*Admission,n(%)*	0				0.007
Direct emer		43 (4.5)	23 (4.6)	20 (4.5)	
Direct observation		1 (0.1)	1 (0.2)	0.0 (0.0)	
Elective		16 (1.7)	7 (1.4)	9 (2.0)	
Ew emer.		434 (45.8)	251 (49.9)	183 (41.1)	
Observation admit		213 (22.5)	88 (17.5)	125 (28.1)	
Surgical same day admission		39 (4.1)	22 (4.4)	17 (3.8)	
Urgent		202 (21.3)	111 (22.1)	91 (20.4)	
*Ethnicity,n(%)*	0				0.016
American Indian/Alaska native		1 (0.1)	1 (0.2)	0.0 (0.0)	
Asian		28 (3.0)	10 (2.0)	18 (4.0)	
Black/African American		150 (15.8)	72 (14.3)	78 (17.5)	
Hispanic/Latino		42 (4.4)	20 (4.0)	22 (4.9)	
Other		143 (15.1)	65 (12.9)	78 (17.5)	
White		584 (61.6)	335 (66.6)	249 (56.0)	
					
*Age, mean (year)*	0	65.5 (14.0)	66.7 (13.3)	64.2 (14.8)	0.005
*Gender,n(%)*	0				0.118
Female		474 (50.0)	264 (52.5)	210 (47.2)	
Male		474 (50.0)	239 (47.5)	235 (52.8)	
*Hypertension,n(%)*	0				0.004
No		774 (81.6)	393 (78.1)	381 (85.6)	
Yes		174 (18.4)	110 (21.9)	64 (14.4)	
*Diabetes,n(%)*	0				0.085
No		555 (58.5)	308 (61.2)	247 (55.5)	
Yes		393 (41.5)	195 (38.8)	198 (44.5)	
*Coronary atherosclerosis,n(%)*	0				0.044
No		666 (70.3)	368 (73.2)	298 (67.0)	
Yes		282 (29.7)	135 (26.8)	147 (33.0)	
*Congestive heart failure,n(%)*	0				0.034
No		620 (65.4)	345 (68.6)	275 (61.8)	
Yes		328 (34.6)	158 (31.4)	170 (38.2)	
*Peripheral vascular,n(%)*	0				0.896
No		901 (95.0)	478 (95.0)	423 (95.1)	
Yes		47 (5.0)	25 (5.0)	22 (4.9)	
*Hyperlipidemia,n(%)*	0				0.273
No		545 (57.5)	298 (59.2)	247 (55.5)	
Yes		403 (42.5)	205 (40.8)	198 (44.5)	
*AMI,n(%)*	0				0.417
No		937 (98.8)	499 (99.2)	438 (98.4)	
Yes		11 (1.2)	4 (0.8)	7 (1.6)	
*Respiratory failure,n(%)*	0				0.297
No		578 (61.0)	315 (62.6)	263 (59.1)	
Yes		370 (39.0)	188 (37.4)	182 (40.9)	
*ESRD,n(%)*	0				<0.001
No		883 (93.1)	489 (97.2)	394 (88.5)	
Yes		65 (6.9)	14 (2.8)	51 (11.5)	
*Laboratory parameters*					
*WBC, mean (SD) (10* ^*9*^ */L)*	13	10.7 (6.1)	9.8 (5.7)	11.7 (6.5)	<0.001
*BUN, mean (SD) (mg/dL)*	0	27.4 (21.2)	22.7 (15.1)	32.7 (25.5)	<0.001
*Creatinine, mean (SD) (mEq/L)*	1	1.4 (1.5)	1.0 (0.7)	1.9 (2.0)	<0.001
*MCH, mean (SD) (pg)*	13	29.4 (2.5)	29.5 (2.4)	29.3 (2.6)	0.338
*MCHC, mean (SD) (g/L)*	13	32.1 (1.6)	32.0 (1.6)	32.1 (1.6)	0.888
*MCV, mean (SD) (fl)*	13	91.8 (6.8)	92.0 (6.6)	91.5 (7.1)	0.222
*Platelet, mean (SD) (10* ^*9*^ */L)*	13	271.7 (155.3)	268.5 (150.6)	275.2 (160.4)	0.511
*RBC, mean (SD) (10* ^*12*^ */L)*	13	3.2 (0.7)	3.2 (0.6)	3.3 (0.7)	0.059
*RDW, mean (SD) (%)*	13	16.1 (2.5)	16.2 (2.6)	16.0 (2.5)	0.396

Abbreviations: Q1: quartile 1; Q2: quartile 2; AG: anion gap; SD: standard deviation; AMI: acute myocardial infarction; ESRD: end-stage renal diseaseesrd; WBC: white blood cell; BUN: blood urea nitrogen; MCH: mean corpusular hemoglobin; MCHC: mean corpusular hemoglobin concerntration; MCV: mean corpusular volume; RBC: red blood count; RDW: red cell distribution width; Ew emer: emergency ward.

**Table 3:** Relationship between anion gap and all-cause mortality in different models.

Variable	Crude model		Model I		Model II		Model III	
	HR (95% CLs)	*P* value	HR (95% CLs)	*P* value	HR (95% CLs)	*P* value	HR (95% CLs)	*P* value
Whole period all-cause mortality								
Anion gap (median), mmol/L								
≤14	1.0 (ref)	0.002	1.0 (ref)		1.0 (ref)		1.0 (ref)	
>14	1.56 (1.18, 2.06)		1.62 (1.21, 2.15)	0.001	1.56 (1.16, 2.09)	0.003	1.47 (1.08, 2.02)	0.016
One-year all-cause mortality								
Anion gap (median), mmol/L								
≤14	1.0 (ref)		1.0 (ref)		1.0 (ref)		1.0 (ref)	
>14	1.47 (1.08, 2.00)	0.014	1.61 (1.17, 2.22)	0.003	1.82 (1.29, 2.56)	0.001	2.18 (1.50, 3.16)	<0.001
Four-year all-cause mortality								
Anion gap (median), mmol/L								
≤14	1.0 (ref)		1.0 (ref)		1.0 (ref)		1.0 (ref)	
>14	1.65 (1.24, 2.18)	0.001	1.77 (1.33, 2.36)	>0.001	1.68 (1.25, 2.25)	0.001	1.52 (1.10, 2.08)	0.01

Notes: the crude model adjusted for none; on the basis of crude model, model I adjusted for age, gender, ethnicity, and skin tone; on the basis of model I, model II adjusted for congestive heart failure, coronary atherosclerosis, diabetes, ESRD, AMI, hyperlipidemia, hypertension, and respiratory failure; on the basis of model II, model III adjusted for creatinine, INR, MCHC, MCH, platelet, RBC, RDW, and WBC. Abbreviations: AG: anion gap; CI: confidence interval; HR: hazard ratio; Ref: reference.

## Data Availability

The clinical data used to support the findings of this study should be obtained upon application to the access of the database, and the database administrator can be contacted at the the public database (MIMIC-IV database V1.0)(https://physionet.org/content/mimiciv/1.0/).

## Supplementary Materials

Supplementary Materials Table S1: predictive power of AG in model III. Figure S1: overall survival curve of high and low AG value groups (AG > 14 and AG ≤ 14) in four time periods.

## References

[1] Naess H., Waje-Andreassen U. (2010). Review of long-term mortality and vascular morbidity amongst young adults with cerebral infarction. *European Journal of Neurology*.

[2] Lendahl U., Nilsson P., Betsholtz C. (2019). Emerging links between cerebrovascular and neurodegenerative diseases-a special role for pericytes. *EMBO Reports*.

[3] Sun F., Liu H., Fu H. X. (2020). Comparative study of intravenous thrombolysis with rt-PA and urokinase for patients with acute cerebral infarction. *The Journal of International Medical Research*.

[4] Sharma V. K., Ng K. W. P., Venketasubramanian N. (2011). Current status of intravenous thrombolysis for acute ischemic stroke in Asia. *International Journal of Stroke*.

[5] Carpenter C. R., Keim S. M., Milne W. K., Meurer W. J., Barsan W. G., Best Evidence in Emergency Medicine Investigator Group (2011). Thrombolytic therapy for acute ischemic stroke beyond three hours. *The Journal of Emergency Medicine*.

[6] Kaste M. (2013). Stroke: advances in thrombolysis. *Lancet Neurology*.

[7] Bray B. D., Campbell J., Hoffman A., Tyrrell P. J., Wolfe C. D. A., Rudd A. G. (2013). Stroke thrombolysis in England: an age stratified analysis of practice and outcome. *Age and Ageing*.

[8] Gill D., Cox T., Aravind A. (2016). A fall in systolic blood pressure 24 hours after thrombolysis for acute ischemic stroke is associated with early neurological recovery. *Journal of Stroke and Cerebrovascular Diseases*.

[9] Kraut J. A., Madias N. E. (2010). Metabolic acidosis: pathophysiology, diagnosis and management. *Nature Reviews. Nephrology*.

[10] Liu X., Feng Y., Zhu X. (2020). Serum anion gap at admission predicts all-cause mortality in critically ill patients with cerebral infarction: evidence from the MIMIC-III database. *Biomarkers*.

[11] Rostami E., Engquist H., Howells T. (2018). Early low cerebral blood flow and high cerebral lactate: prediction of delayed cerebral ischemia in subarachnoid hemorrhage. *Journal of Neurosurgery*.

[12] Martha S. R., Collier L. A., Davis S. M. (2018). Translational evaluation of acid/base and electrolyte alterations in rodent model of focal ischemia. *Journal of Stroke and Cerebrovascular Diseases*.

[13] Agoritsas T., Merglen A., Shah N. D., O’Donnell M., Guyatt G. H. (2017). Adjusted analyses in studies addressing therapy and harm: users' guides to the medical literature. *Journal of the American Medical Association*.

[14] Kraut J. A., Madias N. E. (2007). Serum anion gap: its uses and limitations in clinical medicine. *Clinical Journal of the American Society of Nephrology*.

[15] Oster J. R., Singer I., Contreras G. N., Ahmad H. I., Vieira C. F. (1999). Metabolic acidosis with extreme elevation of anion gap: case report and literature review. *The American Journal of the Medical Sciences*.

[16] Gabow P. A., Kaehny W. D., Fennessey P. V., Goodman S. I., Gross P. A., Schrier R. W. (1980). Diagnostic importance of an increased serum anion gap. *The New England Journal of Medicine*.

[17] Emmett M. (2006). Anion-gap interpretation: the old and the new. *Nature Clinical Practice. Nephrology*.

[18] Yang S. W., Zhou Y. J., Zhao Y. X. (2017). The serum anion gap is associated with disease severity and all-cause mortality in coronary artery disease. *Journal of Geriatric Cardiology*.

[19] Mohr N. M., Vakkalanka J. P., Faine B. A. (2018). Serum anion gap predicts lactate poorly, but may be used to identify sepsis patients at risk for death: a cohort study. *Journal of Critical Care*.

[20] Cheng B., Li D., Gong Y., Ying B., Wang B. (2020). Serum anion gap predicts all-cause mortality in critically ill patients with acute kidney injury: analysis of the MIMIC-III database. *Disease Markers*.

[21] Abramowitz M. K., Hostetter T. H., Melamed M. L. (2012). The serum anion gap is altered in early kidney disease and associates with mortality. *Kidney International*.

[22] Hu B., Cao J., Hu Y., Qin Z., Wang J. (2021). The association between serum anion gap and all-cause mortality in disseminated intravascular coagulation patients: a retrospective analysis. *International Journal of General Medicine*.

[23] Gong F., Zhou Q., Gui C., Huang S., Qin Z. (2021). The relationship between the serum anion gap and all-cause mortality in acute pancreatitis: an analysis of the MIMIC-III database. *International Journal of General Medicine*.

[24] Zhang T., Wang J., Li X. (2021). Association between anion gap and mortality in critically ill patients with cardiogenic shock. *International Journal of General Medicine*.

