# Supplementary material for: The Association between Serum Anion Gap and All-Cause Mortality in Cerebral Infarction Patients after Treatment with rtPA: A Retrospective Analysis
Source: Dis Markers. 2022 May 12;2022:1931818. doi: 10.1155/2022/1931818 (PMC9119778; doi:10.1155/2022/1931818)
Supplement: Supplementary Materials — Table S1: predictive power of AG in model III. Figure S1: overall survival curve of high and low AG value groups (AG > 14 and AG ≤ 14) in four time periods. [file 1931818.f1.docx]

**supplementary material**

**1. Stratified by time period to explore the prediction of AG value > 14 on mortality**

We have specified that the Anion Gap dosage is performed after the rTPA in data extraction part of our manuscript. Furthermore, it would be very important to specify within how long this assay was performed from the rTPA. As shown in Table S1 and Table S2, in Crude Model and Model III, the time factor of measuring AG value was stratified and the predictive ability of AG value on mortality at different time points was calculated. Due to the limited number of patients, we divided the 48 hours into four time periods to analyze the predictive power of AG values. The results are as follows: In Crude Model, we divided the measurement time of AG value of patients after rtPA treatment into four layers. As shown in Figure S1 and Table 2, within 12 to 23 hours，there was a significant difference in mortality between cerebral infarction patients with an AG value > 14 and cerebral infarction patients with an AG ≤ 14 after rtPA treatment ( P = 0.013, HR = 1.905), and similar results were also found within 36 hours to 48 hours (P = 0.037, HR = 2.000).

**Table S1: Predictive power of AG in Model III.**

|  | p-value | HR | Lower 95% | Upper 95% |
| --- | --- | --- | --- | --- |
| 0-11 | 0.171 | 1.534 | 0.831 | 2.833 |
| 12-23 | 0.053 | 1.855 | 0.991 | 3.472 |
| 24-35 | 0.342 | 1.587 | 0.612 | 4.115 |
| 36-48 | 0.24 | 1.629 | 0.722 | 3.676 |

**Table S2: Predictive power of AG in Crude Model.**

|  | p值 | HR | Lower 95% | Upper 95% |
| --- | --- | --- | --- | --- |
| 0-11 | 0.240 | 1.355 | 0.816 | 2.247 |
| 12-23 | 0.013 | 1.905 | 1.145 | 3.175 |
| 24-35 | 0.812 | 0.923 | 0.480 | 1.776 |
| 36-48 | 0.037 | 2.000 | 1.044 | 3.831 |

| 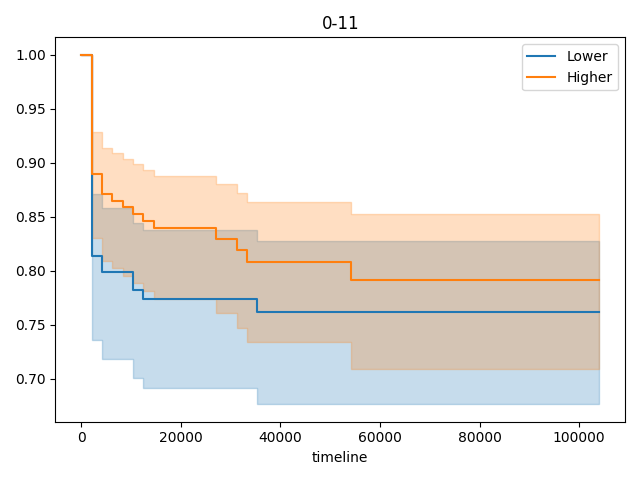 | 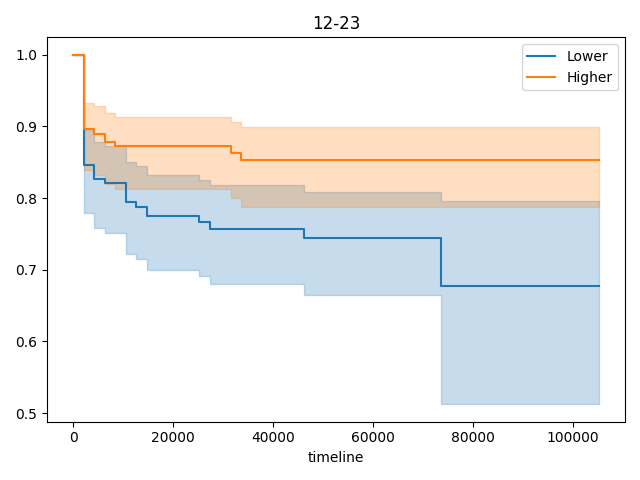 |
| --- | --- |
| 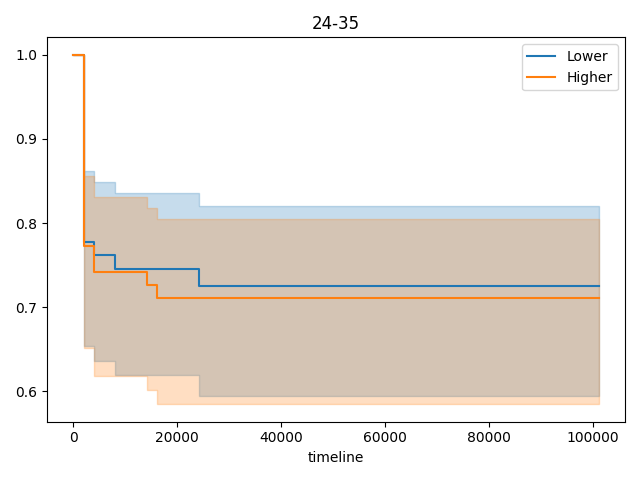 | 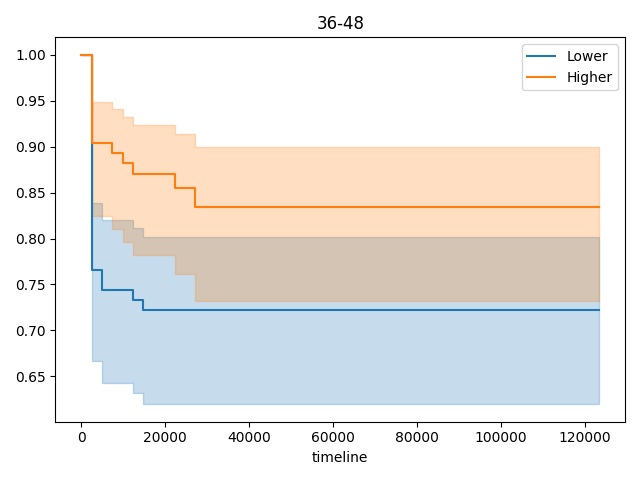 |

**Figure S1: Overall survival curve of high and low AG value groups (AG > 14 and AG ≤ 14) in four time periods.**
